# Sustainable Utilization of Fe(Ⅲ) Isolated from Laterite Hydrochloric Acid Lixivium via Ultrasonic-Assisted Precipitation to Synthesize LiFePO_4_/C for Batteries

**DOI:** 10.3390/ma17020342

**Published:** 2024-01-10

**Authors:** Ziyang Xu, Boren Tan, Boyuan Zhu, Guangye Wei, Zhihui Yu, Jingkui Qu

**Affiliations:** 1 National Engineering Research Center of Green Recycling for Strategic Metal Resources, Institute of Process Engineering, Chinese Academy of Sciences, Beijing 100190, China; zyxu@ipe.ac.cn (Z.X.); brtan@ipe.ac.cn (B.T.); byzhu@ipe.ac.cn (B.Z.); gywei@ipe.ac.cn (G.W.); 2 University of Chinese Academy of Sciences, Beijing 100049, China

**Keywords:** laterite, hydrochloric acid lixivium, ultrasonic, FePO_4_, LiFePO_4_

## Abstract

Ultrasonic-assisted precipitation was employed to sustainably isolate Fe in the hydrochloric acid lixivium of low-grade laterite for the synthesis of battery-grade iron phosphate. The recovery efficiency of Ni and Co exceeded 99%, while the removal efficiency of the Fe impurity reached a maximum of 95%. Precipitation parameters for the selective isolation of Fe (MgO precipitant, pH 1, 70–80 °C) were optimized and used in ultrasonic precipitation experiments. The use of ultrasonic waves in the precipitation process enhanced micromixing by reducing the size of primary grains and mitigating particle agglomeration, thereby significantly improving the purity of the isolated compound and providing high-quality iron phosphate (FePO_4_·2H_2_O). The LiFePO_4_/C composite prepared from as-precipitated FePO_4_ exhibited excellent electrochemical performance, with a discharge capacity of 149.7 mAh/g at 0.1 C and 136.3 mAh/g at 0.5 C after 100 cycles, retaining almost 100% cycling efficiency. This novel and facile method for iron removal from laterite acid lixivium not only efficiently removes excess iron impurities leached due to the poor selectivity of hydrochloric acid, but also enables the high-value utilization of these iron impurities. It enhances economic benefits while simultaneously alleviating environmental pressure.

## 1. Introduction

Electric vehicles offer significant advantages over petrol- and diesel-powered vehicles in addressing the issues of energy shortage and environmental pollution [1]. According to the Global EV Outlook 2023, published by the International Energy Agency, the global electric vehicle market is exhibiting exponential growth; the sales volume of electric vehicles exceeded 10 million units in 2022 [2]. Accordingly, the development of battery technologies, including anode and cathode materials, to power these vehicles is of the utmost importance. Among the various cathode materials, LiFePO_4_-based cathode materials are among the most promising, especially in China, owing to their low cost, high structural and thermal stabilities, and low environmental impact [3,4]. The market volume of LiFePO_4_ batteries was forecast to increase after BYD Company launched the “blade battery”, based on the cell-to-pack technology, in 2020, further increasing the demand for such batteries [2,5,6]. The LiFePO_4_ precursor is one of the most important factors determining the performance of LiFePO_4_ batteries [7]. FePO_4_ is widely used as a LiFePO_4_ precursor owing to its low cost, higher oxidative stability, and superior low-temperature performance compared to other precursors such as iron(II) oxalate, iron(II) acetate, and Fe(OOCCH_3_)_2_ [8,9].

**Figure 1:**
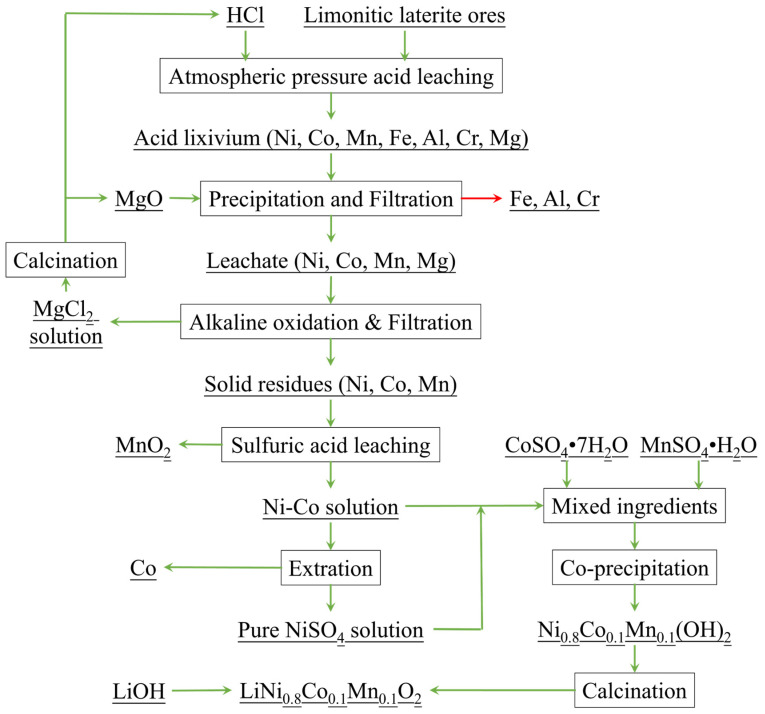
Flow chart illustrating the comprehensive, environmentally sustainable processing of laterite via atmospheric leaching with hydrochloric acid.

FePO_4_ is typically synthesized from analytically or chemically pure salts [10,11]. We previously reported an environmentally sustainable process for the comprehensive treatment of low-grade laterite based on hydrochloric acid atmospheric leaching, which yielded LiNi_0.8_Co_0.1_Mn_0.1_O_2_ cathode materials as the main products (Figure 1) [12,13,14,15]. The processing conditions of our method are milder than those required by high-pressure leaching with sulfuric acid, and the acid medium and MgCl_2_ can be recycled [16,17]; however, the hydrochloric acid atmospheric leaching method exhibits poor selectivity, leading to a substantial ingress of iron ions into the lixivium [18]. The traditional neutralization method for iron removal not only results in significant loss of valuable elements, but also generates a considerable amount of iron slag, thereby wasting resources and causing environmental pollution [19,20,21]. Consequently, the removal of impurity iron in the form of high-purity iron phosphate would facilitate the high-value utilization of waste. Moreover, this synthesis approach offers the advantage of a shorter process compared to synthesis using pure substances; however, the composition of the acidic lixivium of laterite is complex [22]. The conventional co-precipitation method is prone to introduce impurities into the cathode material owing to the high local pH, thereby adversely affecting its electrochemical performance [23].

The important role of mechanical force in the transfer of energy to chemical bonds for driving chemical reactions during manufacturing processes was only recently recognized [24,25]. In 2020, Grzybowski et al. reported that the growth rate of crystals can be significantly accelerated using shear stress generated by fluid dynamics through local stirring (physical effects) rather than by the surface energy effects involved in classical Ostwald ripening (chemical effects) [26]. Furthermore, ultrasound-intensified synthesis effectively enhances the intensity of turbulence and thereby improves micromixing in a reactor to achieve stronger turbulent shear stresses [27]. Sonication can therefore improve the purity and homogeneity of crystals and also mitigate agglomeration [28,29,30]. Moreover, such ultrasonic intensification can be applied in industrial-scale manufacturing processes and material syntheses owing to the rapidity, cost-effectiveness, and environmental friendliness of the process [31].

This study aimed to investigate the impact of ultrasonic enhancement on the removal of iron impurities from atmospheric pressure hydrochloric acid lixivium of laterite using the phosphate method and the synthesis of iron phosphate. Optimal reaction conditions were determined. Under ultrasonic conditions, micro-nano iron phosphate particles were synthesized. Compared to iron phosphate synthesized under conventional conditions, the iron phosphate produced under ultrasonic conditions exhibited higher purity and improved electrochemical properties. This research achieved high-value utilization of iron impurities in low-grade laterite.

## 2. Materials and Methods

### 2.1. Chemicals

All reagents used in this study, including H_3_PO_4_, NaOH, NH_3_·H_2_O, MgO, LiCO_3_, and glucose were of analytical reagent grade. Deionized water was used in all processes. All chemicals were purchased from Shanghai Macklin Biochemical Company (Shanghai, China) and were used directly, without further purification.

The lixivium of low-grade laterite was prepared according to an established procedure [12], which is illustrated in Figure 1. The laterite sample was acquired from Indonesia. The elemental composition of the laterite lixivium is listed in Table 1.

### 2.2. Isolation of Fe from the Hydrochloric Acid Lixivium of Laterite

The precipitation of Fe was performed in a 1 L three-necked flask. H_3_PO_4_ (85 wt%) was added to the hydrochloric acid lixivium of laterite (Fe:PO_4_) in a molar ratio of 1:1 at a specific temperature (50, 60, 70, 80, or 90 °C). The resulting solution was stirred at 350 rpm using a magnetic stirrer. Neutralizers (NaOH, NH_3_·H_2_O, and MgO) were added dropwise/portion-wise to the solution to achieve the target pH (0.5, 1, 1.5, 2, or 2.5). The mixture was stirred for 10 min, and the resultant precipitate was filtered, washed twice with an aqueous hydrochloric acid solution (pH = 2), and dried in an oven at 80 °C for 10 h. In ultrasonic experiments, the lixivium was agitated in an ultrasonic bath (KQ-400DE, Kunshan Ultrasonic Instrument Ltd., Shanghai, China) at an ultrasonic frequency of 40 kHz. In the redissolution experiment, the product was dissolved in dilute hydrochloric acid, and 10% NH_3_·H_2_O was added dropwise at 90 °C until the pH reached 2. The product was then washed twice with deionized water and dried in an oven at 80 °C for 10 h. The retention and removal efficiencies of the specified element in the lixivium were calculated using Equations (1) and (2):

(1) $$\eta_{1}=\frac{c_{2}\times V_{2}}{c_{1}\times V_{1}}\times 100\%$$

(2) $$\eta_{2}=1-\frac{c_{2}\times V_{2}}{c_{1}\times V_{1}}\times 100\%$$

where η_1_ and η_2_ are the retention and removal efficiencies of the specified element (%), respectively; c_2_ is the concentration of the specified element in the filtrate (g/L); V_2_ is the volume of the filtrate (L); c_1_ is the concentration of the specified element in the lixivium used for precipitation (g/L); and V_1_ is the volume of the lixivium (L).

### 2.3. Preparation of the LiFePO_4_/C Composite

The LiFePO_4_/C composite was synthesized from the as-precipitated FePO_4_, commercial Li_2_CO_3_, and glucose via a carbothermic reduction reaction. Precursors were mixed in ethanol and ball-milled for 4 h at a rotation speed of 300 rpm. The solvent was evaporated and the obtained solid was dried in a vacuum oven at 80 °C for 2 h before the precursor mixture was transferred to a tube furnace and calcined at 700 °C for 4 h under a N_2_ atmosphere (heating rate: 5 °C/min). Thereafter, the calcined mixture was naturally cooled to room temperature to yield LiFePO_4_/C as a black powder.

### 2.4. Analysis and Characterization

#### 2.4.1. Material Characterization

The pH of the aqueous solution was measured using a Five Easy Plus pH meter (Mettler Toledo, Greifensee, Switzerland) equipped with an Expert Pro-ISM pH electrode (Mettler Toledo, Greifensee, Switzerland). Standard buffer solutions from Mettler Toledo were used for standardization.

The content of the ions in the filtrate and the elemental composition of the FePO_4_ sample were analyzed using ICP-OES (Optima 8300DV, PerkinElmer, Waltham, MA, USA) with a plasma power of 1150 W while the peristaltic pump was rotated at 100 rpm.

The particle size and morphology of the synthesized cathode material, LiFePO_4_/C, were analyzed via scanning electron microscopy (SEM; JSM-7610F, JEOL, Tokyo, Japan) and TEM (JEM-2100F, JEOL, Tokyo, Japan). The particle size and distribution of the FePO_4_ sample were determined using a nanoparticle size analyzer (Malvern Zetasizer Nano ZS90, Malvern Inc., Malvern, UK). The crystalline phases of the materials were analyzed via powder XRD (Rint-2000, Rigaku, Tokyo, Japan) using Cu *Kα* radiation (40 kV and 40 mA). TG analysis was conducted using the thermogravimetric-differential thermal technique (TGA/DSC3+, Mettler Toledo, Greifensee, Switzerland) at a heating rate of 10 °C/min in a N_2_ atmosphere.

#### 2.4.2. Electrochemical Analysis

Electrochemical properties of the as-prepared LiFePO_4_/C composite were evaluated using CR2032 coin-type cells. The positive electrode was fabricated by mixing the prepared LiFePO_4_/C powder with carbon black (10% *w*/*w*) and polyvinylidene fluoride (10% *w*/*w*) in *N*-methyl pyrrolidinone, and ball-milled to obtain a slurry. The blended slurry was then coated onto an aluminum current collector using an automatic coating dryer, and the electrode was dried in a vacuum drying chamber at 120 °C for 12 h. The test cell consisted of the positive electrode and a lithium foil counter electrode separated by a porous polypropylene film, while the electrolyte consisted of 1 M LiPF_6_ in a 1:1:1 mixture of dimethyl carbonate, ethyl methyl carbonate, and ethylene carbonate. The cell was assembled in a dry glovebox under an argon atmosphere. Electrochemical tests were carried out using a land test system (CT2001A, LAND, Wuhan, China) between 2.5 and 4.1 V at room temperature.

## 3. Results and Discussion

### 3.1. Effects of the Experimental Conditions on the Precipitation of Fe

#### 3.1.1. Effect of the Neutralizer

**Figure 2:**
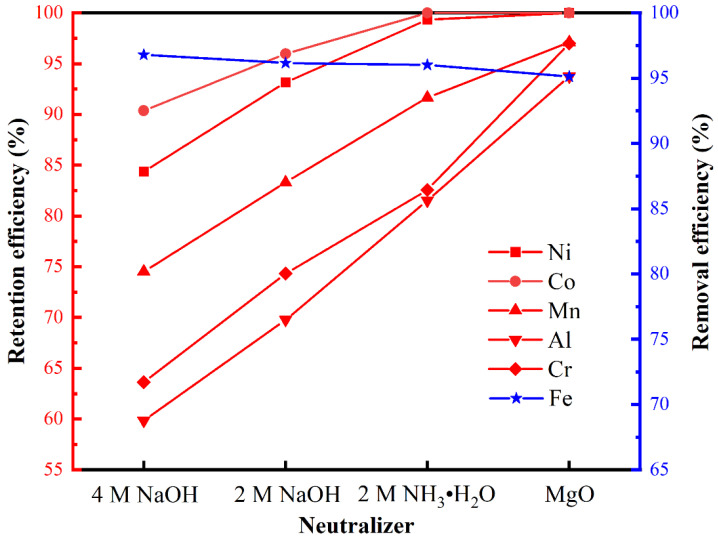
Effect of the neutralizer on the retention and removal efficiencies of specific metals in the laterite lixivium (pH 1, 70 °C) via the phosphate precipitation method.

Figure 2 illustrates the retention efficiencies of Ni, Co, Mn, Al, and Cr, and the removal efficiency of Fe using various neutralizers (pH 1, 70 °C). NaOH and NH_3_·H_2_O are commonly used as precipitants for the synthesis of iron phosphate from chemically or analytically pure iron salts [9,32,33,34]. We therefore employed NaOH and NH_3_·H_2_O for the precipitation of iron phosphate from the laterite lixivium; however, in the case of the complex acid lixivium of laterite, the use of NaOH and NH_3_·H_2_O caused a substantial loss of valuable elements, including Ni and Co (Figure 2). The higher the concentration of NaOH was, the greater the loss of valuable elements. Moreover, the precipitated FePO_4_ contained excessive impurities owing to high local alkalinity arising from the dropwise addition of NaOH and NH_3_·H_2_O. MgO is a by-product of the hydrochloric acid process for laterite (Figure 1). Using a MgO neutralizer, the retention efficiencies of Ni, Co, Mn, Al, and Cr reached 100, 100, 97.1, 93.7, and 97%, respectively. Remarkably, the Fe removal efficiency also reached a high level (≥95%) comparable to that achieved using NaOH and NH_3_·H_2_O neutralizers. Therefore, the optimal choice for the neutralizing agent is MgO. This also achieves the recycling of MgO.

#### 3.1.2. Effect of the Initial pH

**Figure 3:**
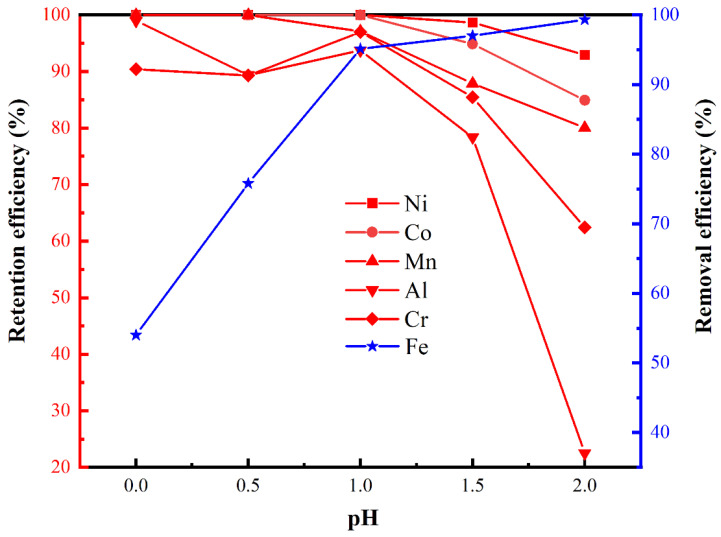
Effect of the initial pH on the retention and removal efficiencies of specific metals in the laterite lixivium (neutralizer: MgO, 70 °C) via the phosphate precipitation method.

As the pH was increased from pH 0 to pH 1, the retention efficiencies of Ni, Co, Mn, Al, and Cr gradually decreased, whereas the removal efficiency of Fe increased rapidly. A further increase in pH, from pH 1 to pH 2, caused the retention efficiencies of Ni, Co, Mn, and especially Al and Cr, to decrease rapidly, whereas the removal efficiency of Fe slowly increased. To ensure low loss rates of Ni and Co, as well as a high removal rate of Fe, pH 1 was shown to be the optimal initial pH for the formation of iron phosphate. As shown in Figure 3, at pH 1, a small amount of Al was incorporated into the precipitate. According to a previous study, Al doping improves the conductivity of LiFePO_4_, which improves both the rate performance and cycle performance of the electrode [23,35].

#### 3.1.3. Effect of the Solution Temperature

**Figure 4:**
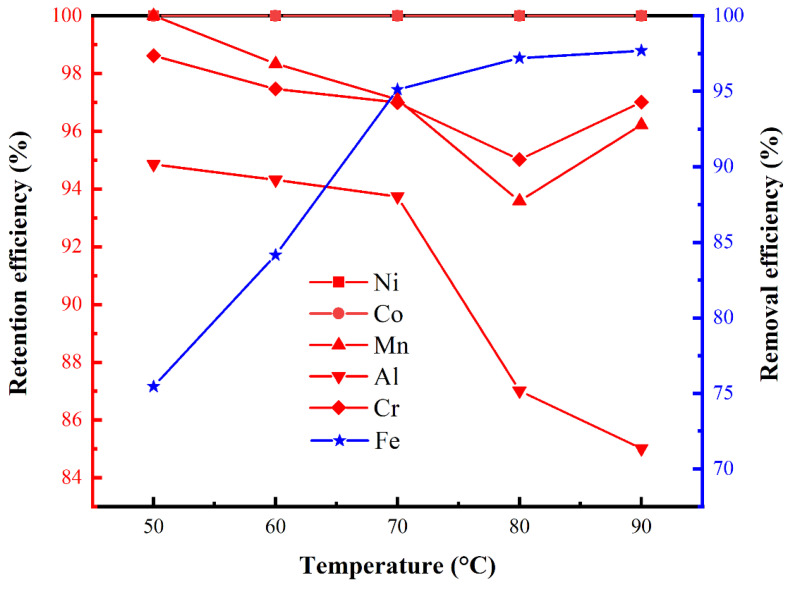
Effect of the solution temperature on the retention and removal efficiencies of specific metals in the laterite lixivium (neutralizer: MgO, pH 1) via the phosphate precipitation method.

The effect of the solution temperature on the retention and removal efficiencies of various metals in the laterite lixivium via the phosphate precipitation method was also investigated. Between 50 and 90 °C, the retention efficiencies of Ni, Co, Mn, and Cr were not significantly affected by the solution temperature (Figure 4). The removal efficiency of Fe increased with temperature; however, the retention efficiencies of Al decreased gradually. This occurred because higher temperatures are more favorable for the crystallization of FePO_4_ and AlPO_4_. Overall, a solution temperature range of 70–80 °C was determined to be optimal for the selective removal of Fe.

In summary, in the absence of ultrasonic irradiation, the optimal conditions for iron phosphate removal from the hydrochloric acid lixivium of laterite were determined to be MgO as the neutralizer, a pH of 1, and temperature = 70 °C.

### 3.2. Ultrasonic-Assisted Precipitation: Effect of Ultrasonic Treatment

**Figure 5:**
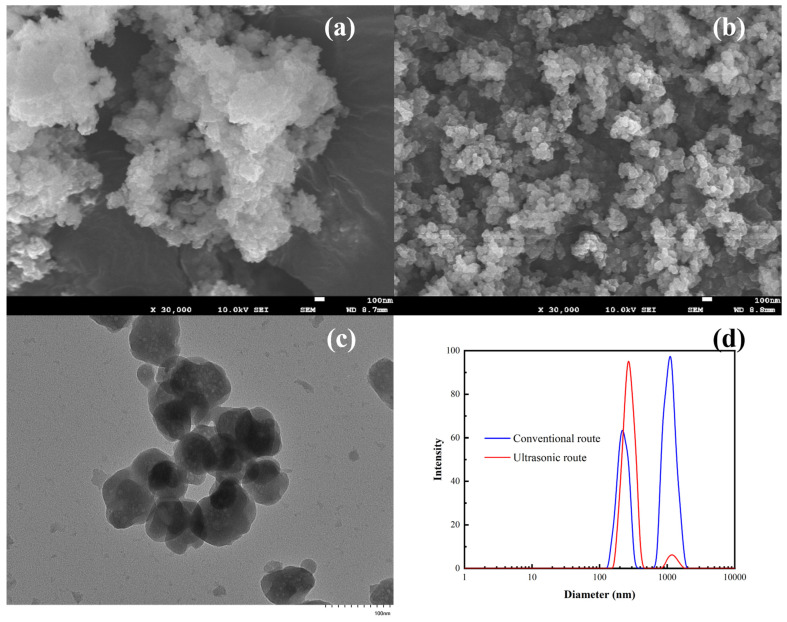
SEM images of iron phosphate precipitated via (**a**) conventional and (**b**) ultrasonic routes. (**c**) TEM image of the iron phosphate sample obtained via ultrasonic-assisted precipitation. (**d**) Particle size distribution of iron phosphate samples obtained using conventional and ultrasonic routes.

The effect of ultrasonic irradiation on particle morphology was studied under optimal conditions. SEM and TEM images of the precipitated iron phosphate revealed that the primary particles of the iron phosphate precipitated using the conventional method (without ultrasonic waves) were larger and agglomerated (Figure 5a). Ultrasonic waves significantly reduced the primary particle size and inhibited agglomeration, leading to micro/nanoparticles of iron phosphate with high dispersibility (Figure 5b,c). The microscale spheroidal secondary grains of iron phosphate measured approximately ~260 nm and consisted of nanospheres with a diameter of ~50 nm. These structures formed because the physicochemical effect of the ultrasound enhanced the maturation of the precipitate and optimized the particle size distribution of the crystals [36]. The observed size distribution of the nanoparticles (Figure 5d) was consistent with SEM and TEM results.

The chemical compositions of iron phosphate samples obtained using conventional and ultrasonic-assisted precipitation was quantitatively determined via ICP-OES (Table 2). The ultrasonic-assisted precipitation method significantly reduced the content of impurities, particularly Al and Cr, because the ultrasonic waves enhanced local turbulence, preventing localized increases in pH caused by the neutral agent, thereby inhibiting the precipitation of impurities [28]. The Fe/P molar ratio is an important index of the quality of iron phosphate; thus the Fe/P molar ratios of the products prepared using conventional and ultrasonic-assisted precipitation are provided in Table 2.

**Figure 6:**
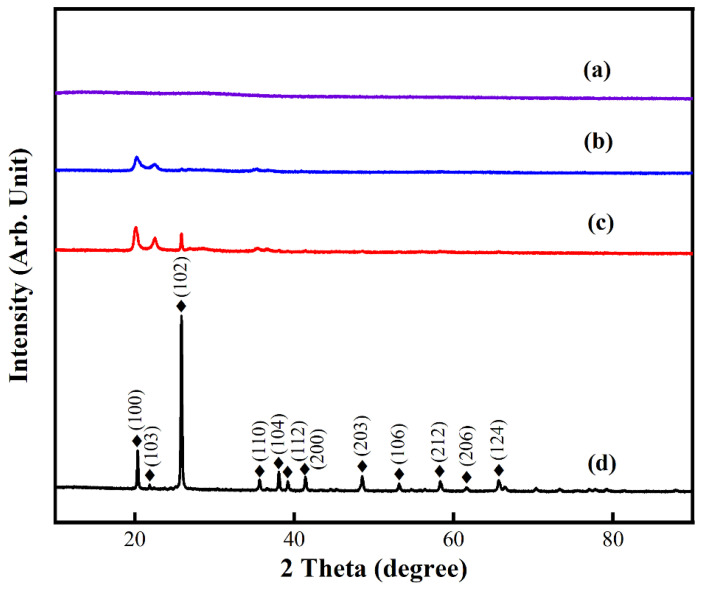
X-ray diffraction patterns of FePO_4_ precipitates obtained using conventional and ultrasonic routes: (**a**) as-precipitated sample obtained using the conventional method; (**b**,**c**) sample precipitated via the conventional route using NH_3_·H_2_O and MgO, respectively, and calcined at 650 °C for 4 h; and (**d**) sample precipitated via the ultrasonic route using MgO and calcined at 650 °C for 4 h.

The phase purity of particles obtained using the two methods was analyzed using XRD (Figure 6). Before calcination (curve a), the diffractogram of the product showed no strong peaks, indicating that FePO_4_ synthesized via wet chemistry was amorphous; therefore, the effect of different processing conditions on the phase purity of the precipitated product was investigated after calcining it at 650 °C for 4 h. Using the conventional method with a NH_3_·H_2_O precipitant (curve b), diffraction peaks were observed at approximately 20.12°, 21.3°, 22.8°, and 29.46°, indicating the formation of AlPO_4_ (JCPDS card No. 00-050-0302). With MgO as the precipitant (curve c, conventional method), the XRD pattern showed the presence of a mixture of FePO_4_ and AlPO_4_. However, when ultrasonic waves were applied for agitation using an MgO precipitant (curve d), the intensity and sharpness of the XRD peaks increased significantly, suggesting that the calcined product had a higher crystallinity; the observed peaks could be indexed to the anhydrous hexagonal structure of FePO_4_ (JCPDS card No. 29-0715, a = 5.035 Å, b = 5.035 Å, c = 11.245 Å). No impurity-related diffraction peaks were observed in the XRD pattern, confirming the high purity of the precipitated FePO_4_.

**Figure 7:**
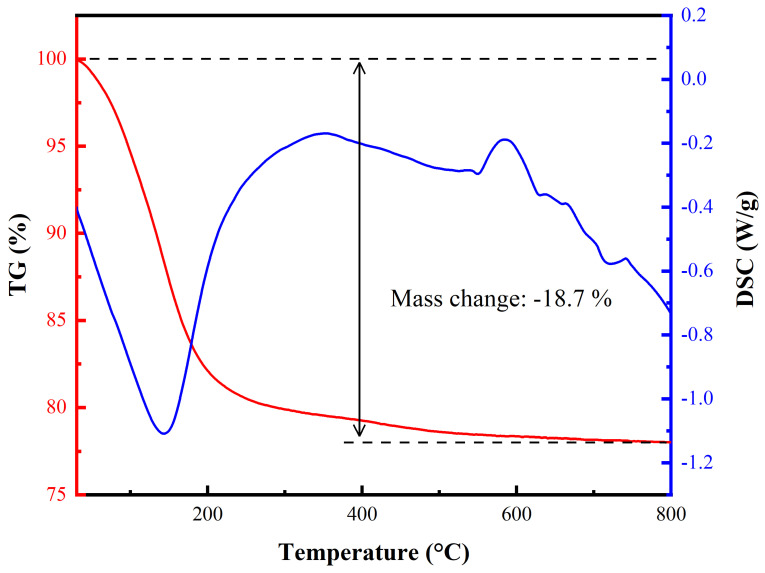
TG/DSC curves of iron phosphate obtained via the ultrasonic precipitation method.

The TG/DSC curve of the iron phosphate precipitated via the ultrasonic method using MgO (Figure 7) reveals an apparent weight loss of ~18.7% between ambient temperature and 500 °C, corresponding to the loss of crystal water. This is similar to the theoretical weight loss expected for FePO_4_·2H_2_O (19.27%, 2.00 H_2_O), indicating that the general formula of the precipitated product is FePO_4_·2H_2_O. The corresponding DSC curve exhibits a clear endothermic peak at ~140 °C. The TG curve shows no significant mass loss after 500 °C, indicating that both the crystal water and adsorbed water were completely removed. In addition, the DSC curve shows two exothermic peaks, at 585 and 740 °C, which were attributed to the phase transition from amorphous to crystalline FePO_4_ [37,38].

**Figure 8:**
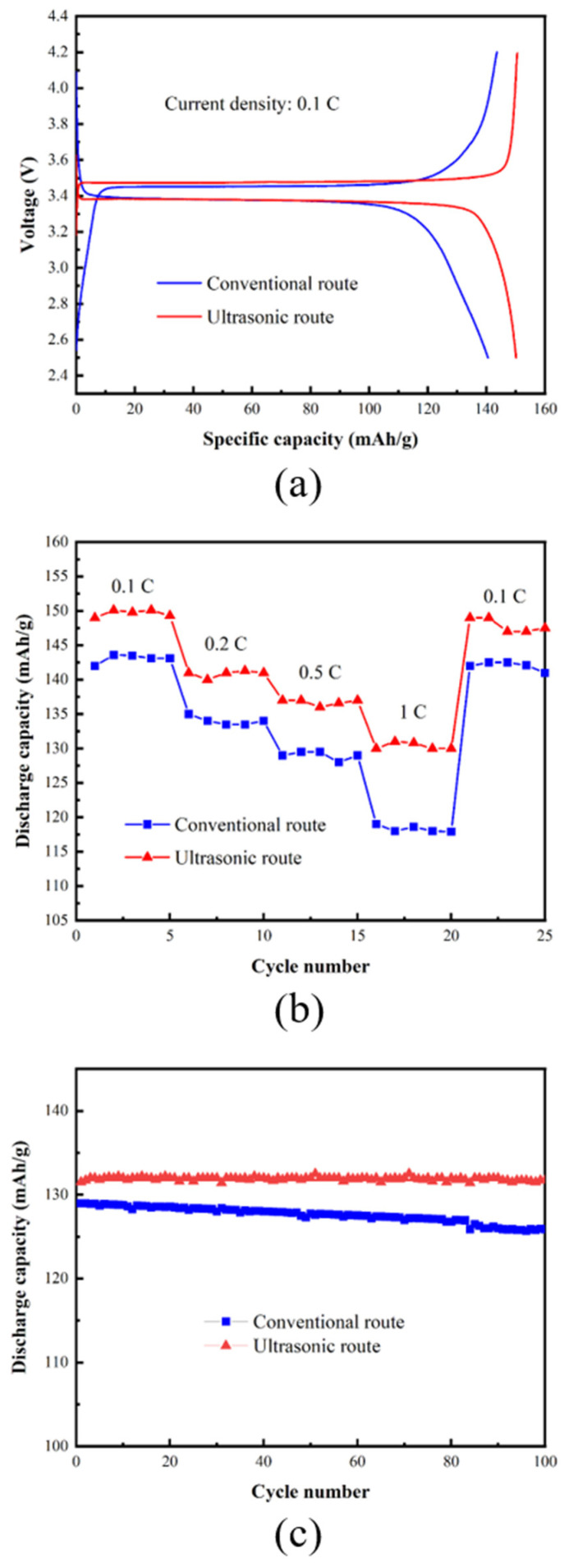
Electrochemical performances of iron phosphate samples precipitated via conventional and ultrasonic routes. (**a**) Initial charge/discharge curves at 0.1 C, (**b**) rate performance at various rates, and (**c**) cycling performance at 0.5 C over 100 cycles.

Figure 8 compares the electrochemical performance of LiFePO_4_/C composites prepared from iron phosphate samples precipitated via conventional and ultrasonic methods, following redissolution and refinement. The discharge curves of both samples at 0.1 C exhibit a long and flat voltage plateau at ~3.4 V (Figure 8a), reflecting the two-phase redox reaction between FePO_4_ and LiFePO_4_, and the polarization in both samples was small. The discharge capacities of the electrode prepared from the iron phosphate precipitated via the ultrasonic route were 149.7, 139.8, 140.6, and 136.3 mAh/g at 0.1, 0.2, 0.5, and 1 C, respectively (Figure 8b), which were higher than those of the electrode prepared from the iron phosphate precipitated using the conventional route (141.1, 127.5, 114.7, and 103.3 mAh/g at 0.1, 0.2, 0.5, and 1 C, respectively). Furthermore, at 0.5 C, the capacity retention of electrodes fabricated using iron phosphate samples obtained using conventional and ultrasonic routes were uniformly 100% (Figure 8c).

## 4. Conclusions

We presented an environmentally sustainable and facile route for the selective removal of Fe from the hydrochloric acid lixivium of low-grade laterite via ultrasonic-assisted precipitation, which produces battery-grade iron phosphate in a single step. The following conclusions were drawn from this study:

1. The use of MgO as a neutralizing agent limits the loss of main elements, such as Ni and Co, caused by high local pH. MgO can also be recycled without introducing other impurities. Both the pH and temperature of the solution significantly affect the recovery rate of main elements and the removal rate of Fe from the lixivium of laterite. The optimum pH and temperature for Fe precipitation are 1 and 70–80 °C, respectively.
2. The application of ultrasonic waves during precipitation reduces the size of primary grains and inhibits particle agglomeration. In addition, the level of impurities in the Fe precipitate decreased, thereby increasing the phase purity of the isolated iron phosphate. This may be attributed to enhanced micromixing arising from the increased local turbulence caused by ultrasonication. The chemical formula of the precipitated iron phosphate is FePO_4_·H_2_O.
3. Battery-grade FePO_4_ was isolated from lixivium, and ultrasonic treatment improved the electrochemical performance of LiFePO_4_ prepared from as-precipitated FePO_4_.

## Figures and Tables

**Table 1:** Elemental composition of the laterite lixivium and theoretical initial pH for metal phosphate precipitation.

	Ni	Co	Fe	Mn	Mg	Al	Cr	Ca
Concentration (g/L)	0.739	0.077	34.667	0.623	0.617	1.757	0.733	0.357
Phosphate pKsp	31.32	34.69	21.89	31.21	23.90	18.24	21.52	28.70
Initial precipitation pH	2.40	2.00	0.07	2.42	3.58	1.61	0.74	2.86

**Table 2:** Compositions (wt%) and Fe/P molar ratios of iron phosphate samples precipitated via conventional and ultrasonic routes.

Sample	Fe	P	Ni	Co	Mn	Al	Cr	Fe/P Molar Ratio
Conventional precipitation	30.45	13.85	0	0.001	0.012	0.164	0.068	1.22
Ultrasonic-assisted precipitation	28.49	13.77	0	0	0.0003	0.044	0.016	1.15

## Data Availability

The data supporting the article’s findings are available from the corresponding author upon reasonable request.
